# Supplementary material for: A regressive analysis of the main environmental risk factors of human echinococcosis in 370 counties in China
Source: PLoS Negl Trop Dis. 2024 May 14;18(5):e0012131. doi: 10.1371/journal.pntd.0012131 (PMC11125469; doi:10.1371/journal.pntd.0012131)
Supplement: Attachment — Submitted filename: Responses to the reviewers.docx [file pntd.0012131.s001.docx]

**Answer to reviewers**

**Reviewer #1:**

**Comment 1. Is the study design appropriate to address the stated objectives?**

**Answer:** Yes. Our study was aiming at exploring the influence of environmental factors on the prevalence of human echinococcosis, and to provide reference for the formulation of prevention and control measures of echinococcosis in the future. Our design takes into account that the relative changes in environmental factors are not significant, and collects environmental factor variables and other related variables at the county scale. With the progress of central transfer payment projects, almost all counties have undergone population screening with a coverage rate of over 90%. Perform pathogen testing (ELISA for fecal testing) on more than 20 dogs (including domestic and unowned dogs) in each administrative village (community) using dissection or arecoline hydrobromide catharsis method, and all dogs in villages (communities) with less than 20 dogs are tested. Calculate the infection rate of echinococcus in dogs. We used the ratio of cumulative discovered patients in each county to all high-risk populations in that county as the incidence rate, collected a sufficient number and representative samples at the county scale, and finally determined the impact and magnitude of environmental factors through multiple regression analysis. So, we believe that our research design can achieve the established goals.

**Comment 2. It is necessary to detail the methodology used for diagnosis in dogs. Environmental samples or samples collected directly from the host were analyzed?**

**Answer:** We have added explanations in the article. The diagnosis of dogs is mainly carried out through fecal antigen detection reagents. Our unit is responsible for evaluating the reagents, and we will select the reagents with excellent reliability and stability in the evaluation and recommend them to be used in various epidemic counties. With the advancement of the central transfer payment project, the number of stray dogs in epidemic areas is relatively small, so the samples for dog feces testing are all from domestic dogs. Require the collection of fresh dog fecal samples with the help of the head of household, in order to minimize cross infection caused by other parasites caused by the environment.

**Comment 3. The diagnostic methodology to estimate the prevalence in humans is not described. It is very important to discriminate the age range, since cases in people under 15 years of age refer to active cycles of CE transmission.**

**Answer:** Each epidemic county has basically carried out population screening projects, with a coverage rate of over 90%. Incidence rate of residents' echinococcosis: Each township (town, street) carries out abdominal ultrasound examination for local residents aged 2 years and above, diagnoses according to industry standards, and determines whether they are ill with B-ultrasound assisted serological examination. Calculate the prevalence of human echinococcosis.

The age grouping of patients is a good suggestion, but in this study, we do not investigate the temporal changes in prevalence. Our article mainly studies the relationship between environmental characteristics and the degree of transmission of echinococcosis. Our research objective is to investigate the spatial differences in the distribution of echinococcosis caused by these environmental factors, so we used the cumulative incidence rate calculated from all patients. The incidence rate of people under 15 years old is more representative of the current environmental transmission risk, while older patients represent previous transmission risks. So in future longitudinal studies on the trend of transmission risk, we will conduct detailed grouping analysis of patient age.

**Comment 4. Does the analysis presented match the analysis plan?**

**Answer:** Yes. We can ensure the correctness of all statistical methods.

**Comment 5. The samples analyzed by ELISA were confirmed by WB? If so, these results must be reported. It is known that there are cross reactions with other helminths, this denotes a strong limitation for the interpretation of the results, due to the possibility of false positives.**

**Answer:** The results of ELISA do not require WB confirmation. We test dog fecal sample reagents every year and recommend a comprehensive reagent for use in various provinces. So, we believe that although false positive results exist, this reagent is the most practical among all reagents. Moreover, all epidemic counties use the same reagent, so their results are comparable.

**Comment 6. "Extreme cold weather often occurs in this season, which may cause the intermediate host animals to freeze to death in the wild, directly increasing the field transmission cycle".**

**This can also imply the death of the metacestode**

**Answer:** No. Usually, dead intermediate host animals have larvae that can survive for a long time. Before being frozen to death, it is highly likely to be eaten by the final host wild animals, and we believe that this can still increase the field transmission cycle

**Comment 7. "Studies have found that when NDVI is below 0.5 or above 0.7 in spring, the prevalence of echinococcosis gradually increases, and when NDVI is between 0.5 and 0.7, the prevalence gradually decreases"**

**Please explain better the cause of this variation.**

**Answer:** This conclusion requires more rigorous argumentation, and we have deleted it in the article.

**Comment 8. The authors should discuss the lack of specificity of the technique used to detect cases in definitive hosts (copro-ELISA). Although this work provides valuable information on the epidemiology landscape, the concept of one health is not presented, addressed or discussed.**

**Answer:** We have added an explanation of the lack of specificity in fecal ELISA detection technology. We have added a discussion on overall health. Analyzed the relationship between population, host animals, and environment from the perspective of overall health. The high prevalence area of echinococcosis is mainly located in the Qinghai Tibet Plateau, which has significant environmental characteristics. Due to this specific environment, the production mode in the region is mainly animal husbandry, and the socio-economic situation is relatively backward. The basic health care conditions of the population are not perfect, and the awareness of disease prevention is relatively lacking. Multiple factors together lead to the spatial distribution characteristics of echinococcosis.

**Comment 9. Due to there are cross-reactions with other taenids, how can you ensure that positive results only belong to echinococcus spp.?**

**Answer:** In order to ensure the accuracy of the results and minimize the false positive rate, we conduct annual testing on dog fecal sample reagents and recommend the most comprehensive reagent currently available on the market for use in various provinces. So we do not deny the existence of false positive results, but this reagent is the most practical among all reagents, and all popular counties use the same reagent, so their results are comparable, which is the most important point. If PCR detection is used in the entire epidemic area, due to the large sample size of 20 per village, most epidemic counties do not have PCR detection conditions, which cannot be achieved in terms of feasibility.

**Comment 10. The** **manuscript needs severe revisions regarding terminology. It is mandatory to unify terms such as:** **definitive host, intermediate host, echinococcus spp. eggs, E. granulosus s.l., etc. I suggest consulting "International consensus on terminology to be used in the field of echinococcoses" Vuitton et al., 2020** [**https://doi.org/10.1051/parasite/2020024**](https://doi.org/10.1051/parasite/2020024)

**Answer:** We have revised the relevant terminology in the manuscript

**Comment 11. This work describes the relationships between environmental elements and the risk of human echinococcosis. Although this work responds to a one-health approach, this factor is not adequately addressed. The manuscript needs a thorough review of language and terminology. The authors must report limitations when calculating the prevalence in definitive hosts, due to the lack of specificity of the technique used (copro-ELISA). Likewise, it is necessary to provide more information on how the prevalence data in humans were obtained.**

**Answer:** We have provided detailed and serious answers to the previous questions. We have supplemented and revised the relevant article content. Thank you for your review.

**Reviewer #2:**

**Comment 1. The authors have stated in the Introduction that the prevalence of echinococcosis and environmental factors have strong spatial autocorrelation. But when determining the relationship of environmental factors and the risk of echinococcosis in negative binomial regression model, the spatial autocorrelation is not well considered and controlled in model fitting.**

**Answer:** You may have misunderstood the content. The introduction points out that ‘epidemic factors of echinococcosis are not only related to personal exposure, but also closely related to environment itself’, rather than a strong spatial autocorrelation between the two. Spatial autocorrelation refers to the single attribute variable for different observation objects, and in this article, it refers to the incidence of echinococcosis. Therefore, when modeling, a negative binomial regression model is fitted, which is aimed at the situation where the dependent variable has spatial autocorrelation.

**Comment 2. Reasons of LST, NDVI, and** **elevations groupings are not described.**

**Answer:** The elevation is a fixed value, and we extracted the average elevation at the county scale, describing the distribution of different elevations in each popular county without grouping it.

The satellite footage we downloaded has a 7-day cycle, from which we extracted LST and NDVI, and calculated the average values of LST and NDVI for each month. The regression model of LST and LST and NDVI for each month shows that there is multicollinearity between adjacent months, which can interfere with the results. Considering the significant climate differences in each season of the epidemic area, we calculated the average LST and NDVI for each quarter on a seasonal basis, in order to group the LST and NDVI into the model. These contents are included in the ‘Acquisition of environmental data’ of the article.

Taking into account the consistent trend of LST across all seasons, which decreases with increasing elevation, we included the average annual LST in the negative binomial regression model. These contents are included in the ‘Fitting negative binomial model’ of the article.

**Comment 3.** **Social factors are also significantly involved in the epidemic process of human echinococcosis, and why it is not controlled here?**

**Answer:** There are two reasons why social factors is not controlled here. Firstly, the main objective of this study is to research the relationship between environmental factors and the incidence of echinococcosis, and social factors are not our main focus. Secondly, considering the high correlation between social factors and the environmental factors that this study focuses on, social factors were not included in the model during modeling to avoid overfitting and maintain the simplicity of the model.

We have conducted research on the impact of social factors, and the link is as follows ”http://1.202.226.196:8888/rwt/PUBMED/https/P75YPLUPMNSGTLUPNSXT65UJNAYGP55X/pmc/articles/PMC9587573/”

**Comment 4. Betas and its 95% CIs from regression models are insufficient for public health practitioners to get the epidemiological meaning of the association in magnitude, and I would like to suggest to use its exponential form here.**

**Answer:** Thank you for your reminder. This is a very good question. We have adjusted the expression of the table to better understand the actual results of the model.

**Comment 5. The authors concluded that the main environmental risk factors were altitude, LST, and NDVI associated with echinococcosis, which was not sufficiently supported by the results.**

**Answer:** We have revised the wording of this conclusion based on the results section. The current conclusion is ”The prevalence of human echinococcosis is affected by environmental factors, such as annual average LST, spring NDVI, summer NDVI, average elevation.”

**Comment 6. The tables and figures need arrangement and modifications to make the results more concise.**

**Answer:** Thank you for your suggestion. We have tried our best to make the tables and numbers more concise.

**Comment 7. Present work investigated several environmental factors and its association with the prevalence of human echinococcosis in epidemic areas of mainland China. In reviewing the work, several potential limitations and deficiencies in terms of study design come to attention. It is necessary to address these issues to enhance the rigor and reliability of the study's findings.**

**Answer:** Dear reviewer, thank you for this important comment. We have made modifications and additions to all the questions raised by the two editors, and resolved these issues.

**Comment 8. Findings were difficult to adjudicate due to the poor grammar and structure problem. The paper needs English language professional editing. And laborious and problematic sentences are not limited to the following sentences: “40% of CE cases and 90% of AE cases worldwide occur in China and the disease burden of CE and AE in China respectively accounts for 40% and 90% of the global disability-adjusted life years (DALYs)” in page 8 and “The lower the LST, the higher the risk of the population” in page 21.**

**Answer:** We have consulted professionals whose native language is English to revise the content of the article.

**Comment 9. The current title was not self-explanatory and its study design are recommended to added.**

**Answer:** We have modified the title to “A regressive analysis of the main environmental risk factors of human echinococcosis in 370 counties, in China”.
